# Nitroaromatic Antibiotics as Nitrogen Oxide Sources

**DOI:** 10.3390/biom11020267

**Published:** 2021-02-12

**Authors:** Allison M. Rice, Yueming Long, S. Bruce King

**Affiliations:** Department of Chemistry and Biochemistry, Wake Forest University, Winston-Salem, NC 27101, USA; ricea@wfu.edu (A.M.R.); longy17@wfu.edu (Y.L.)

**Keywords:** nitroaromatic antibiotics, nitric oxide (NO), nitrite, nitroxyl (HNO), reactive nitrogen species (RNS), infectious diseases, metronidazole

## Abstract

Nitroaromatic antibiotics show activity against anaerobic bacteria and parasites, finding use in the treatment of *Heliobacter pylori* infections, tuberculosis, trichomoniasis, human African trypanosomiasis, Chagas disease and leishmaniasis. Despite this activity and a clear need for the development of new treatments for these conditions, the associated toxicity and lack of clear mechanisms of action have limited their therapeutic development. Nitroaromatic antibiotics require reductive bioactivation for activity and this reductive metabolism can convert the nitro group to nitric oxide (NO) or a related reactive nitrogen species (RNS). As nitric oxide plays important roles in the defensive immune response to bacterial infection through both signaling and redox-mediated pathways, defining controlled NO generation pathways from these antibiotics would allow the design of new therapeutics. This review focuses on the release of nitrogen oxide species from various nitroaromatic antibiotics to portend the increased ability for these compounds to positively impact infectious disease treatment.

## 1. Introduction

**Figure 1:**
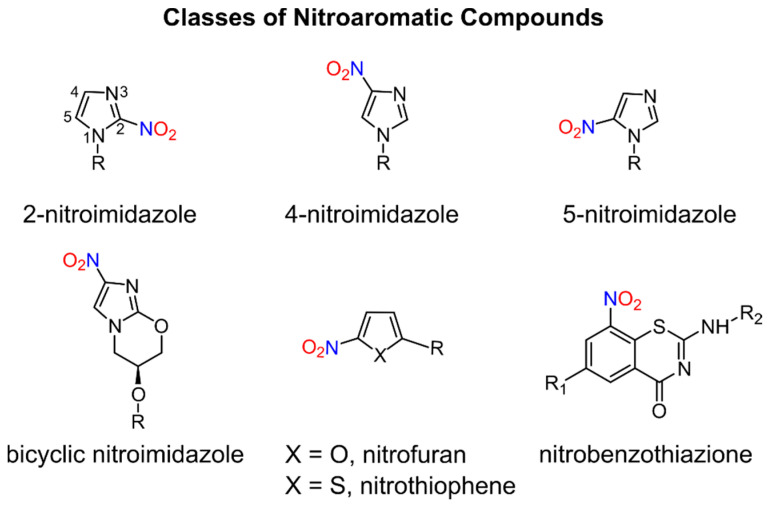
General classes of biologically active nitroaromatic compounds.

Nitroaromatic compounds contain one or more nitro groups directly bonded to an aromatic group (Figure 1) [1].

Numerous nitro heteroaromatic compounds, especially those containing imidazole and furan or thiophene groups as depicted in Figure 1, demonstrate biological activities allowing for their clinical use as antibiotics and are discussed in this review.

The chemical and physical properties of the nitro group (−NO_2_) including its electron-withdrawing ability, polarity, size, ability to form hydrogen bonds and redox properties [1,2] contribute to its key role in the action of many drugs, especially antimicrobial agents. Nitroaromatic compounds find use in the treatment of a wide variety of infectious diseases for both bacterial infections, including tuberculosis (TB), and parasitic infections such as giardiasis, trichomoniasis, human African trypanosomiasis (HAT), Chagas disease and leishmaniasis, which are increasingly becoming global health threats [3]. Despite the antibacterial and antiparasitic properties of nitroaromatic antibiotics, drugs containing nitro groups often demonstrate mutagenicity and unacceptable toxicity profiles, which have hindered further development of this drug class. The potent biological activity of these compounds drives the search for non-mutagenic and selectively toxic nitroaromatic antibiotics that kill the infectious agent without damaging host cells [1,4]. In addition, repurposing existing nitroaromatic antibiotics that have been proven safe represents a less risky and cost-efficient strategy in the search of new and effective drugs to treat neglected tropical diseases (NTD) [3].

**Scheme 1:**
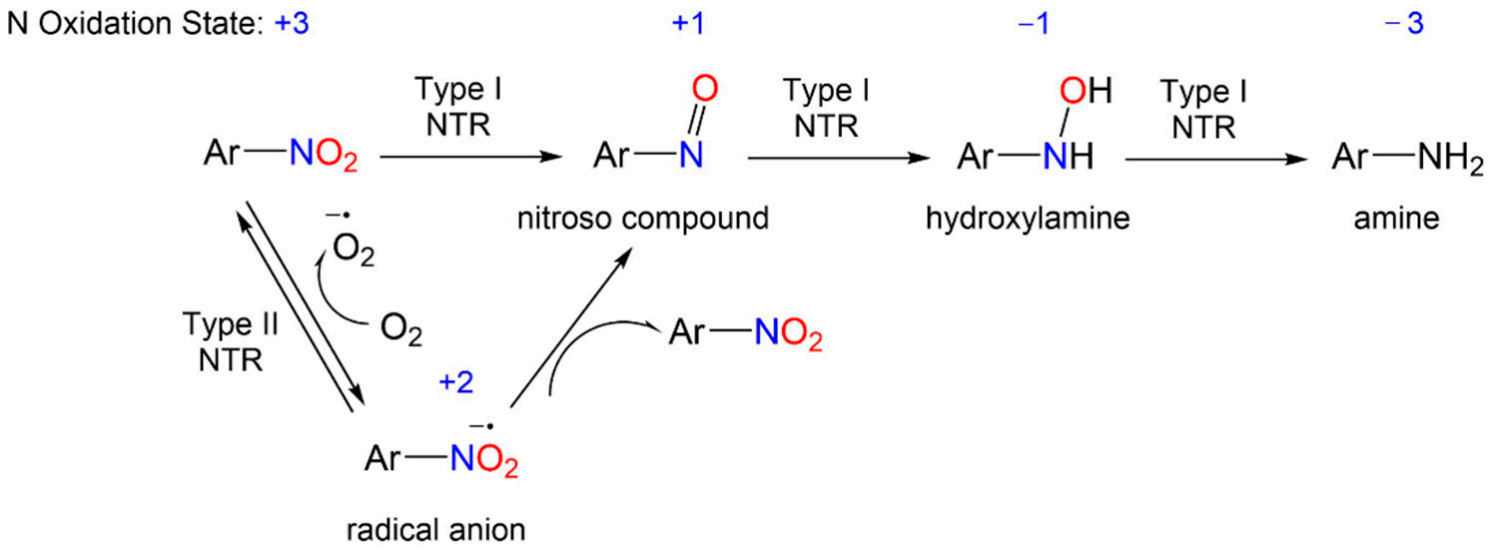
General reduction mechanism of nitroaromatic compounds.

The redox biochemistry of the nitro group plays a central role in the biological activity of nitroaromatic antibiotics. These compounds require reductive bioactivation for antimicrobial activity with the reduction being carried out by nitroreductases (NTRs) of type 1 or 2 (or type I or II, Scheme 1) that possess reducing power greater than the reduction potential of the nitroaromatic compound. Numerous organism-specific nitroreductases exist as well as other systems including pyruvate/ferredoxin oxidoreductase and hydrogenases that are capable of catalyzing nitro group reduction [5,6,7,8,9,10].

These enzymes catalyze nitroaromatic compound (N oxidation state = +3) reduction using flavin mononucleotide (FMN) or flavin adenine dinucleotide (FAD), and nicotinamide adenine dinucleotide (NADH) or nicotinamide adenine dinucleotide phosphate (NADPH) as reducing agents [11,12]. Type I NTR (NTR1), which is oxygen-insensitive, catalyzes the reduction of the nitro group through a series of two-electron reductions yielding a nitroso group (N oxidation state = +1), a hydroxylamine (N oxidation state = −1) and ultimately an amine (N oxidation state = -3) (Scheme 1). On the other hand, type II NTR (NTR2), which is oxygen-sensitive, catalyzes a one-electron reduction of nitroaromatic compounds, resulting in a nitro radical anion (N oxidation state = +2). The nitro radical anion can disproportionate to the starting nitroaromatic compound and a nitroso species or react with oxygen to give the nitroaromatic compound and the reactive superoxide anion (Scheme 1). The uncoupling of NADPH-derived reducing equivalents from normal metabolism or the formation of reactive radical, nitroso or hydroxylamine intermediates, which can react with other biomolecules, provides a potential mechanism for nitroaromatic antibiotics activity. The formation of these reactive species, including superoxides, coupled with the presence of various nitroreductases in different target organisms, also likely plays a role in the observed toxicity, mutagenicity and lack of selectivity of nitroaromatic antibiotics. Thus, the nitro group in these prodrugs triggers the formation of reactive intermediates upon reduction that either directly mediate the biological effects or further fragment to other reactive species.

**Scheme 2:**
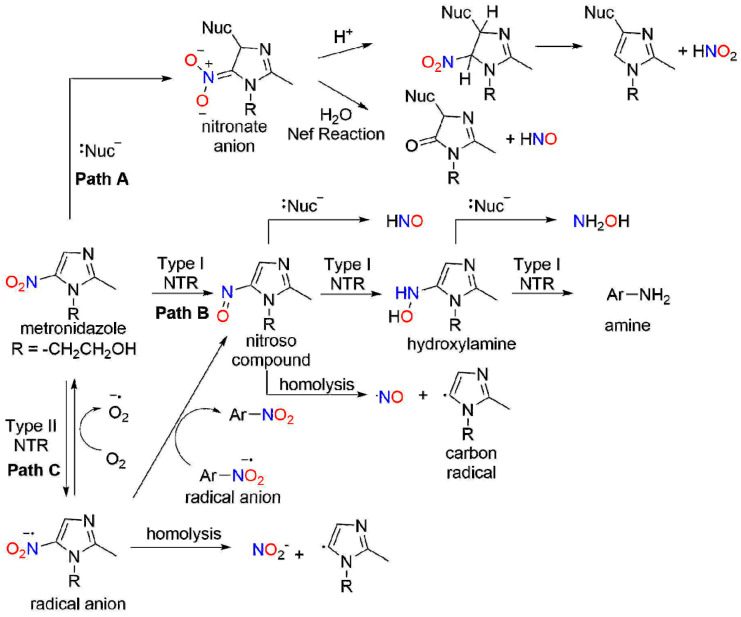
Potential chemical mechanisms for release of nitrogen oxide species from metronidazole.

The bioreductive metabolism of nitroaromatic compounds also provides an opportunity for them to act as prodrugs of reactive nitrogen species (RNS), particularly various nitrogen oxides [2]. Scheme 2, described more fully below, shows possible chemical pathways of RNS release from metronidazole, as an example nitroaromatic antibiotic. These nitrogen oxides include nitrite (NO_2_^−^, N oxidation state = +3), nitric oxide (NO, N oxidation state = +2) and nitroxyl (HNO, N oxidation state = +1), redox relatives with important implications in biology. For example, NO_2_^−^_,_ a stable solid salt, undergoes a one-electron reduction to yield NO (+2 oxidation state), which is a colorless, radical gas at normal temperature and pressure. Nitrite finds extensive use as a food preservative and acts as a source of NO under reductive conditions, demonstrating a biological profile similar to NO. Since the discovery of NO as a biological signaling agent formed by the nitric oxide synthase (NOS)-catalyzed oxidation of L-arginine, its potential as an antibacterial agent has attracted great interest [13]. As an endogenously produced free radial, NO kills bacteria through both oxidative and nitrosative stressors, which include lipid peroxidation, nitrosation of membrane proteins and DNA damage through reactions with RNS [14,15,16,17,18]. The use of NO and NO-releasing materials as antimicrobial agents has been reviewed [19]. The multiple antibacterial mechanisms of NO decrease the risk of bacterial resistance, providing another benefit of NO/redox-based therapies [19,20]. Further one-electron reduction and protonation of NO yields HNO, a highly reactive species that rapidly dimerizes and dehydrates to nitrous oxide (N_2_O, e.g., laughing gas) [21]. Despite the structural similarity to NO, HNO shows a distinct biological profile compared with NO based on different chemistries with heme proteins and thiols [22,23,24,25,26,27,28]. Various spectroscopic, electrochemical and chemiluminescent techniques exist to measure NO_2_^−^, NO or HNO, which makes it possible to track and monitor their presence [29]. Nitro group reductive metabolism with nitrogen oxide release defines nitroaromatic antibiotics as prodrugs increasing the likelihood of identifying active non-toxic lead compounds [1,4].

Based on the cytotoxic and antibacterial properties of NO and the known reductive metabolism of nitroaromatic compounds, the possibility that NO release mediates a portion of the activities of nitroaromatic antibiotics exists. Scheme 2 depicts potential chemical mechanisms for nitrogen oxide release from metronidazole, a common 5-nitroimidazole antibiotic. Nucleophilic aromatic substitution to the carbon β with the nitro group yields a Meisenheimer-type complex that includes a nitronate anion resonance form (Scheme 2, Path A). Nitrous acid elimination directly produces nitrous acid/NO_2_^−^ and a new nucleophile-substituted imidazole. Alternatively, nitronate anion hydrolysis would yield HNO (Nef reaction) and a lactam-like derivative (Scheme 2, Path A). These reactions produce nitrogen oxides from nitro aromatic compounds through direct nucleophilic addition without a nitro group reduction. Scheme 2, Path B shows a direct two-electron NTR1 reduction of the nitro group of metronidazole to produce a nitroso intermediate. Nucleophilic substitution as shown above would be expected to yield HNO and a nucleophile-substituted imidazole. Alternatively, homolytic bond cleavage of the nitroso compound would give NO and an imidazole-based carbon radical (Scheme 2, Path B). Further NTR1 reduction of the nitroso intermediate gives a hydroxylamine derivative that could also undergo a nucleophilic substitution to yield hydroxylamine and a nucleophile-substituted imidazole. Loss of water from the hydroxylamine generates a reactive nitrenium ion capable of triggering ring fragmentation or reaction with nucleophiles. Scheme 2, Path C shows a direct one-electron NTR2 reduction of the nitro group of metronidazole to give a radical anion intermediate. Homolytic bond cleavage of this radical anion yields NO_2_^−^ and the same imidazole-based carbon radical as shown above. The radical anion also could also undergo further redox chemistry (disproportionation or reaction with oxygen to give superoxide anion and starting metronidazole). Overall, Scheme 2 shows a number of plausible chemical pathways for the production of nitrogen oxides (NO_2_^−^, NO, HNO and hydroxylamine) from nitro-substituted aromatic compounds. Differences in the redox potential of the nitro groups and the heteroaromatic rings may direct reactivity between Paths A and C and provide a structural control element for the design of new compounds.

Understanding the scope and mechanisms of nitrogen oxide release could portend new avenues in the therapeutic development of anticancer, antibacterial, anti-tubercular and antiparasitic agents [2], as well potential treatments for NTD Many nitroaromatic antibiotics demonstrate evidence of nitrogen oxide species release including 5-nitroimidazoles (e.g., metronidazole, tinidazole and related compounds), 2-nitroimidazoles (azomycin, misonidazole and benznidazole), bicyclic 4-nitroimidazoles (e.g., PA-824, OPC-67683, TBA-354 and related compounds) and 2-nitrofurans/2-nitrothiophenes (e.g., nifurtimox). This review specifically highlights the metabolism and release of nitrogen oxide species from these nitroaromatic antibiotics and is divided into sections detailing nitroimidazoles, nitrofurans/nitrothiophenes and other nitroaromatic antibiotics and their biological activity, nitrogen oxide species-releasing ability and mechanism of release. Other reviews focus on nitroaromatic antibiotic drug development or clinical treatment of TB and other infectious diseases [3,30,31].

## 2. Nitroimidazoles

### 2.1. 5-Nitroimidazoles

**Figure 2:**
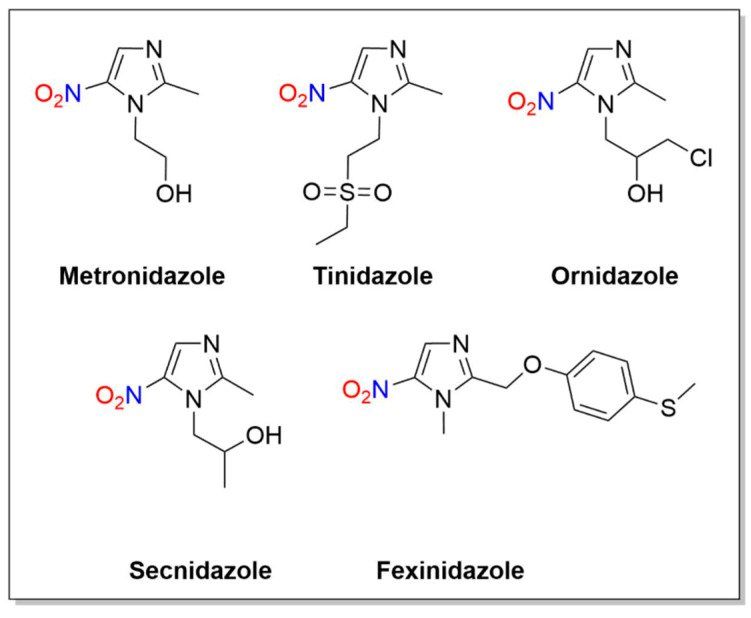
Structures of metronidazole, tinidazole, ornidazole, secnidazole and fexinidazole.

Metronidazole (1-(2-hydroxyethyl)-2-methyl-5-nitroimidazole) is an antibacterial drug containing a 5-nitroimidazole core similar to known antibiotics including tinidazole, ornidazole, secnidazole and fexinidazole (Figure 2).

#### 2.1.1. Metronidazole

Metronidazole has been used in clinical practice since the 1950s to treat anaerobic bacterial and parasitic infections, and usage continues for trichomoniasis, amebiasis, giardiasis and vaginosis, as well as pelvic inflammatory disease, colorectal surgical and *H. pylori* infections (Figure 2) [32,33,34]. In addition to its broad treatment ability, metronidazole is cost-effective with an acceptable adverse effect profile and undiminished antimicrobial activity [32]. The mechanism of action of metronidazole is based on four phases: (I) cell entry, (II) reduction of the nitro group, (III) cytotoxic effects arising from the reduction products, (IV) removal of inactive end products [32,33,35].

**Scheme 3:**
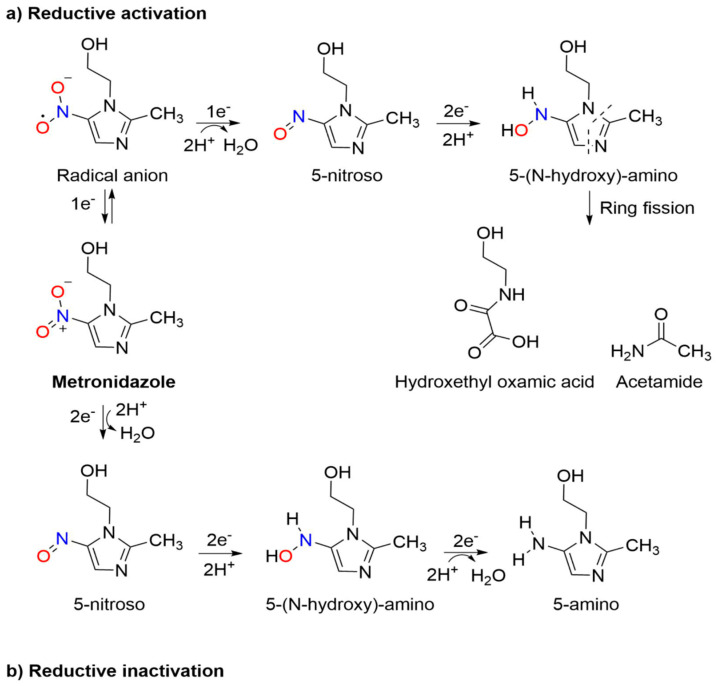
Two routes of metabolic reduction of metronidazole: (**a**) reductive activation and (**b**) reductive inactivation [33].

Metronidazole acts as a prodrug, remaining inactive until reduction, and the metabolism, structure–cytotoxicity and resistance mechanisms have been recently reviewed.^33^ The metabolic reduction of metronidazole occurs through two routes: (1) reductive inactivation, leading to the inactive 5-amino derivative and (2) reductive activation, resulting in nitro group reduction, leading to cleavage of the imidazole ring giving acetamide and an oxamic acid derivative (Scheme 3) [33]. Metabolism requires enzymes with a sufficient reductive potential, such as pyruvate/ferredoxin oxidoreductase, which generates adenosine triphosphate (ATP) through the oxidative decarboxylation of pyruvate. Other systems including hydrogenases and various nitroreductases also reduce metronidazole [36,37]. Reductive inactivation proceeds through the reduction of the nitro group to the non-toxic amino derivative, an oxygen-insensitive process that occurs in two-electron steps with a total of six electrons consumed [33,38]. Reductive activation of the nitro group leads to activation of metronidazole through imidazole fragmentation that mediates cytotoxicity. This process begins with the formation of a nitro radical anion, capable of leading to the generation of reactive oxygen species and oxidative damage to the cell, and consumes four electrons. It is thought to occur in a series of one- or two-electron steps leading to ring-fission, forming potentially cytotoxic derivatives (acetamide and an oxamic acid, Scheme 3) [33,39,40,41,42].

The formation of acetamide and the oxamic acid from metronidazole reduction does not account for all three nitrogen atoms of metronidazole. It remains unclear whether the cytotoxic activity of metronidazole results from these reduction pathways or subsequent fragmentation of the imidazole ring and metabolite formation [33]. 

**Scheme 4:**
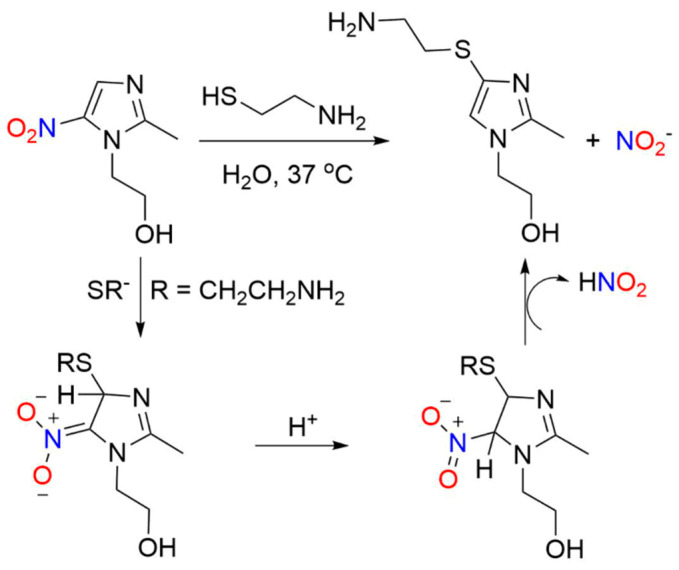
Reaction of metronidazole with 2-aminoethanethiol to yield 4-[(2-aminoethyl)thio]-2-methylimidazole-1-ethanol and nitrous acid (nitrite) [43].

Early pivotal studies confirm the nitrogen oxide release from metronidazole and structurally similar compounds upon nucleophilic substitution of the nitro group of 5-nitroimidazoles by thiols [43]. Metronidazole reacts with 2-aminoethanethiol to yield 4-[(2-aminoethyl)thio]-2-methylimidazole-1-ethanol and nitrous acid (nitrite) (Scheme 4) [43]. These reactions produce isomeric imidazole products and demonstrate a pH dependence that lead to a plausible mechanistic proposal of direct thiol addition to the nitroimidazole forming a Meisenheimer-type complex that liberates nitrous acid while restoring aromaticity [43]. These results clearly show direct nitrogen oxide release from metronidazole in the form of NO_2_^−^ and define a substitution mechanism that does not require direct nitro group reduction (Scheme 2, Path A and Scheme 4) [43]. 

Other earlier work showed that glutathione S-transferase-catalyzed transfer of glutathione (GSH) to over fifty 2-substituted nitrofuran derivatives yields GSH conjugates in rat and human liver homongenates [44]. Under these conditions, 2-nitrofuran derivative conversion to the corresponding GSH conjugate occurs with the release of NO_2_^−^, as determined spectrophotometrically [44]. Both alkali lability and pH considerations affect reactivity, and 16 out of the 52 tested compounds yield GSH conjugates and release NO_2_^−^, providing direct evidence of nitrogen oxide release from these nitroaromatic antibiotics [44]. Interestingly, metronidazole does not generate NO_2_^-^ in this system [44].

A study of metronidazole’s effect on tumor radiosensitization through iron-catalyzed reactions with thiol groups provides evidence of formation of a metronidazole–cysteine conjugate [45]. Metronidazole treatment of tumor-bearing mice before X-ray exposure results in a reduction in the radiation dose necessary to control tumors, possibly due to the ability of the nitro group to act as a strong radical oxidant [45]. Based on absorption experiments, metronidazole rapidly reacts with an iron–cysteine complex to give a purple colored reduced drug (radical anion)–iron–cysteine complex [45]. Despite the formation of this conjugate, no evidence of nitrogen oxide species formation was reported [45]. Subsequent to this report, electron paramagnetic resonance (EPR) spectroscopic studies showed the mixture of metronidazole, cysteine and ferrous ions yields iron–cysteine–NO complexes, indicating NO release from metronidazole under these reducing conditions [46]. The suggested mechanism for NO formation involves loss of NO_2_^-^ from the radical anion of the nitro group of metronidazole (Scheme 2, Path C). Taken together, these results clearly indicate the ability of metronidazole to release both NO_2_ and NO through distinct reactions (addition of thiols to the heteroaromatic ring or direct reduction of the nitro group with thiols/iron).

#### 2.1.2. Metronidazole–NO Donor Hybrids 

**Figure 3:**
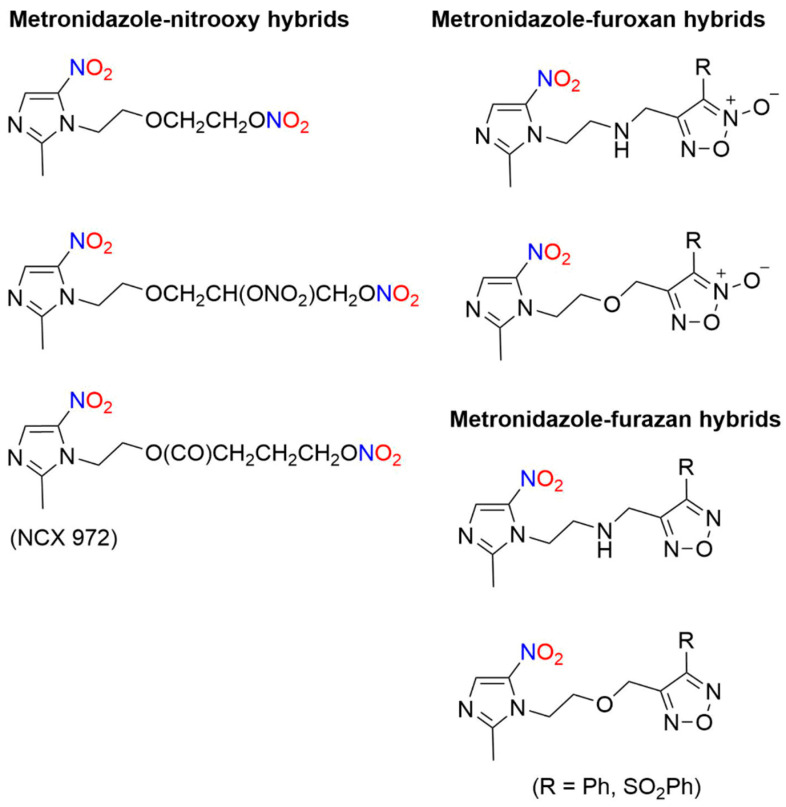
Structures of various metronidazole–NO donor hybrids [47,48].

The intentional addition of a NO-donating group to metronidazole gives metronidazole–NO donor hybrids that release NO. A library of NO–donor metronidazole hybrids was synthesized for use as therapeutics to treat issues related to *H. pylori* infections, such as gastric ulcers and extra gastrointestinal disorders [47]. Based on the NO-donating ability of furoxans, hybrid molecules were constructed with metronidazole or its amino analog joined through spacers to furoxan moieties, as well as a group of compounds containing one or two nitrooxy groups (Figure 3) [47]. Furazans (1,2,5-oxadiazoles) served as controls lacking the ability to release NO [47]. These hybrid compounds showed good in vitro activity against various *H. pylori* strains with a lower minimum inhibitory concentration (MIC) than metronidazole [47]. In terms of the NO-donating properties, a significant difference in potency between the corresponding furazan/furoxan pairs was not observed, indicating that the N-oxide substituent present in the furoxan hybrids that donates NO and induces oxidative stress was not the major determinant of activity [47].

NCX 972 ([4-(nitrooxy)butanoic acid 2-methyl-5-nitroimidazole-1-ethyl ester) combines the basic metronidazole structure with a nitrate ester, another known NO donor moiety (Figure 3) [48]. The in vitro effects of metronidazole were compared to NCX 972 in trophozoite cells for the treatment of amoebiasis against *Entamoeba histolytica*, a protozoan parasite [48]. NCX 972 releases NO into amoebic trophozoites as measured by the diaminofluorescein-2 diacetate (DAF-2DA) dye detection system that reacts with an oxidized NO species to generate irreversible DAF fluorescence [48]. In comparison, NCX 972 displays both higher and faster cell killing activity than metronidazole in *E. histolytica*, which correlates with NO release into the cells [48]. These data could portend new avenues for drug derivatization with NO-releasing moieties to already established drugs.

**Figure 4:**
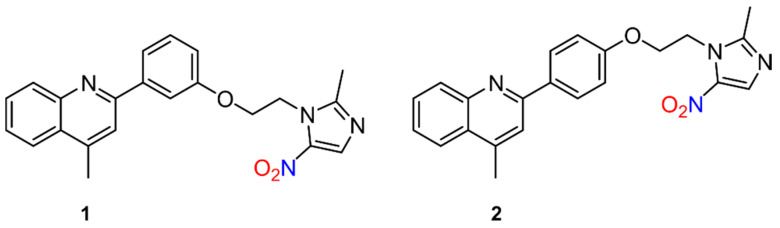
Quinoline–metronidazole derivatives active against visceral leishmaniasis (VL) [49].

Similarly, the mechanism of action of quinoline–metronidazole hybrids was recently investigated against visceral leishmaniasis (VL) [49]. The rationale for merging the two compounds stems from the use of metronidazole to combat experimental VL, especially in combination with other drugs [50,51,52,53], along with the activity of quinolines and their derivatives against in vitro and in vivo forms of experimental and cutaneous VL [54,55]. To determine the anti-leishmanial potency of quinoline−metronidazole derivatives, inhibition of promastigotes and intracellular amastigotes of *Leishmania donovani* was tested at various concentrations with various regioisomeric compounds [49]. Compounds 4-methyl-2-(3-(2-(2-methyl-5-nitro-1H-imidazol-1-yl)ethoxy)phenyl)quinolone (**1**) and 4-methyl-2-(4-(2-(2-methyl-5-nitro-1H-imidazol-1-yl)ethoxy)phenyl)quinolone (**2**) (Figure 4) were found to be the most active as determined by a luciferase and 3-(4,5-dimethylthiazol-2-yl)-2,5-diphenyltetrazolium bromide (MTT) assay, while **2** most effectively inhibited parasites in the liver and spleen of infected mice [49]. To examine NO release in relation to leishmanicidal action, the DAF-2DA dye assay was utilized, which implies local NO formation in living cells at the intracellular level, and revealed **2** leads to oxidative stress through the enhancement of both ROS and NO generation [49]. While these compounds do not contain traditional NO donor groups, these results further show nitrogen oxide species release from 5-nitroimidazoles similar to metronidazole. 

Metronidazole possesses a relatively simple structure with functionality positioned to allow further elaboration. Eckmann, Sharpless and co-workers demonstrated the structural versatility of the metronidazole core by generating an extensive library of diverse 5-nitroimidazole-based metronidazole derivatives [56]. Preparation of a metronidazole-derived azide allows copper (I)-catalyzed azide-alkyne cycloadditions (“click reactions”) that give a large number of metronidazole derivatives in high yield under simple and mild conditions. Several of these new compounds show increased antimicrobial activity (>100-fold compared to metronidazole) while being active and non-toxic in animal infection models, as well as showing the ability to overcome different forms of drug resistance [56]. Such work shows the synthetic potential for designing the next generation of metronidazole-based novel scaffolds that include nitrogen oxide-generating groups for anti-infective disease treatments. 

#### 2.1.3. Tinidazole

Tinidazole (1-(2-ethylsulfonylethyl)-2-methyl-5-nitroimidazole), an antibiotic similar to metronidazole in structure, was first introduced in 1969, followed by its approval by the US Food and Drug Administration (FDA) in 2004 for the treatment of bacterial infections such as vaginosis and trichomoniasis, as well as a therapy for parasitic infections such as giardiasis and amebiasis (Figure 2) [57,58].

Due to the structural similarity to metronidazole (e.g., the 2-(hydroxyl)ethyl group is replaced by a 2-(ethylsulfonyl)ethyl moiety in the 5-nitroimidazole backbone, Figure 2), tinidazole was suspected to have an analogous mechanism of action, as well as potential for NO donation. Direct current (DC) polarographic experiments confirm the ability of tinidazole to donate NO through nitro group hydrolysis to generate NO_2_^−^, determined by the spectrophotometric Griess assay [59]. The chemical investigations showing NO release from tinidazole were further confirmed with biological data showing that tinidazole activates soluble guanylate cyclase (sGC), indicative of NO formation, in a comparable manner to metronidazole [59].

#### 2.1.4. Secnidazole

Secnidazole (1-(2-methyl-5-nitroimidazole-1-yl)propan-2-ol) is an antibacterial drug containing a 5-nitroimidazole core, similar to metronidazole (Figure 2). Secnidazole shows clinical efficacy against anaerobic bacteria and parasitic infections such as trichomoniasis and amebiasis. The mechanism of action of secnidazole is based on the ability of the nitro group to accept electrons [60]. Different voltammetric techniques (e.g., cyclic voltammetry and controlled potential electrolysis) to study secnidazole reduction in aqueous media reveal two well-defined reduction waves in acidic media, while at higher pHs, the two waves overlap [60]. In acidic media, secnidazole is reduced to hydroxylamine in a four-electron process, with the nitroso derivative as an intermediate [60]. Given the structural similarity to metronidazole, the release of NO_2_^−^/NO from secnidazole would be expected but has not been definitively confirmed.

#### 2.1.5. Ornidazole

Ornidazole (1-(2-hydroxy-3-chloropropyl)-2-methyl-5-nitroimidazole) is a 5-nitroimidazole antibiotic used to treat infections caused by both anaerobic bacteria and protozoa including giardiasis, Crohn’s disease and *Dientamoeba fragilis* (Figure 2) [61,62,63]. The antibiotic ability is very similar to metronidazole, and the selective toxicity against bacteria cells depends on reduction of the nitro group to the radical anion [64]. Similar to secnidazole, the reduction of ornidazole was studied using normal pulse polarography and reverse pulse polarography [64]. Depending on pH, ornidazole demonstrates one or two waves in aqueous solution, with the first wave corresponding to a four-electron reduction to hydroxylamine and the second wave corresponding to the subsequent reduction to an amine [64]. 

A Zn^2+^ complex of ornidazole (Zn(Onz)_2_Cl_2_) decreases the formation of the radical anion species [65]. While the antibacterial activity of ornidazole depends on the formation of the reduced radical anion and other reduced reactive species (e.g., hydroxylamine and nitroso), prolonged exposure to the radical anion reduces tissue oxygenation and generates a superoxide and peroxide, leading to neurotoxicity [65]. Thus, controlling the generation of the radical anion becomes crucial. The Zn^+2^ ornidazole complex was examined for radical anion formation and efficacy against *E. histolytica* compared to ornidazole [65]. Using xanthine oxidase to reduce the nitro group of ornidazole, a change in the absorption spectra is observed as evidence of irreversible nitro group reduction [65]. The Zn^+2^ ornidazole complex showed controlled reduction to the radial anion with no significant decrease in absorbance [65]. In addition, the MIC, against *E. histolytica* after 48 h exposure, was lower for the Zn^+2^ ornidazole complex compared to ornidazole [65]. These results support the idea that the complex has a similar or even better efficacy in treating infection while maintaining a lessened cytotoxicity [65]. The role of nitrogen oxides in the biological activity of ornidazole or its Zn^+2^ complex has not been investigated but would be expected based on the structural similarity to metronidazole.

#### 2.1.6. Fexinidazole

Fexinidazole (1-methyl-2-[(4-methylsulfanylphenoxy)methyl]-5-nitroimidazole), a 5-nitroimidazole derivative developed by Hoechst AG, was shown by the Drugs for Neglected Diseases initiative (DNDi) to demonstrate activity against *Trypanosoma brucei gambiense* and *T. b. rhodesiense* in 2005 and is now undergoing phase III trials for HAT and Chagas disease (Figure 2) [66].

Following NTR1 reduction and bioactivation, fexinidazole produces reactive amine species that are indirectly toxic and mutagenic to trypanosomes, while two principal metabolites, fexinidazole sulfoxide and fexinidazole sulfone, result in the anti-trypanosomal activity [67,68]. Given that the genome of the leishmania parasites contains a homologous nitroreductase gene to the trypanosomes, experiments were undertaken to test the ability of fexinidazole to treat VL [69]. The results indicated that overexpression of the leishmanial homolog of this nitroreductase in *L. donovani* increases sensitivity to fexinidazole by 19-fold, which leads to the idea that a similar mechanism is occurring in both parasites [69]. Direct release of nitrogen oxides from fexinidazole has not been demonstrated, but such activity could play a role in the observed biological activities.

### 2.2. 2-Nitroimidazoles

**Figure 5:**
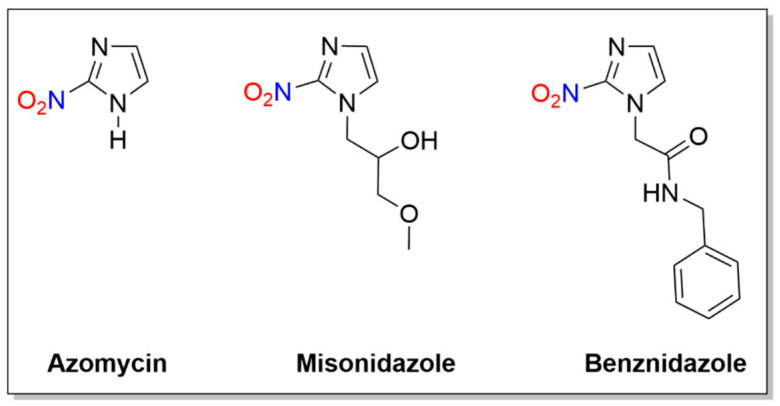
Structures of azomycin, misonidazole and benznidazole.

Some 2-nitroimidazole compounds discussed in the context of this review are azomycin, misonidazole and benznidazole (Figure 5). Unlike metronidazole, these reductions of 2-nitroimidazoles do not generate nitrogen oxides and all of the nitrogen atoms remain in the observed products.

#### 2.2.1. Azomycin

**Scheme 5:**
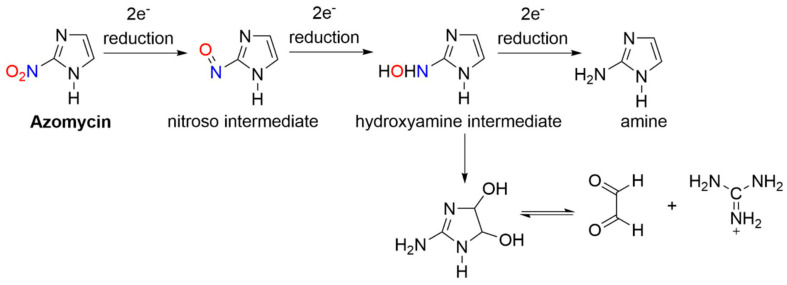
Anaerobic reduction of 2-nitroimidazoles (e.g., azomycin) [71].

Azomycin (2-nitroimidazole) was isolated in 1953 from a culture liquid containing a strain of bacteria similar to *Nocardia mesenterica* and first synthesized in 1965 (Figure 5) [70]. In early studies of 2-nitroimidazoles, chemical reduction of azomycin and misonidazole gave 2-aminoimidazole (e.g., six-electron) and 2-(hydroxylamino)imidazole (e.g., four-electron) products [71,72,73]. NMR spectroscopic studies showed the major products of the reduction of azomycin were the *cis:trans* isomers of 1-substituted 2-amino-4,5-dihydro-4,5-dihydroxyimidazolium ions in 70–85% yield [71]. These products likely form from the condensation of glyoxal and guanidine decomposition products (Scheme 5) [71]. 

The four-electron-reduced hydroxylamine is more stable in acidic media and converts to glyoxal, a highly reactive dialdehyde, while at a neutral pH, hydroxylamine is not observed, but the decomposition to glyoxal competes with the further two-electron reduction to the amine [71]. The finding of glyoxal adducts is in accordance with the identification of a glyoxal derivative from the urine of a patient being administered misonidazole [74]. Reduction of azomycin to a glyoxal precursor is biologically important due to the known interaction between glyoxal and cellular compounds. 

#### 2.2.2. Misonidazole

Misonidazole (1-methoxy-3-(2-nitro-1H-imidazol-1-yl)propan-2-ol) (Figure 5) was examined for NO release by EPR spectroscopy in the presence of cysteine and ferrous ions and compared to metronidazole [46]. While metronidazole produced a three-line EPR spectrum indicative of NO release and formation of an NO–iron–cysteine complex, misonidazole did not produce the same spectrum, indicating the lack of NO formation [46]. Optical spectroscopy revealed nitro group reduction [46], leading to the conclusion that the reduced nitro group remains attached to the imidazole ring that is in accordance with a previous report that only 1% NO_2_^−^ is observed during the electrolytic reduction of misonidazole [75]. This body of evidence shows a clear difference in nitrogen oxide release between 2- and 5-nitroimidazoles during reduction, indicating the nitro group position in nitroaromatic compounds influences the decomposition pathway.

#### 2.2.3. Benznidazole

**Scheme 6:**
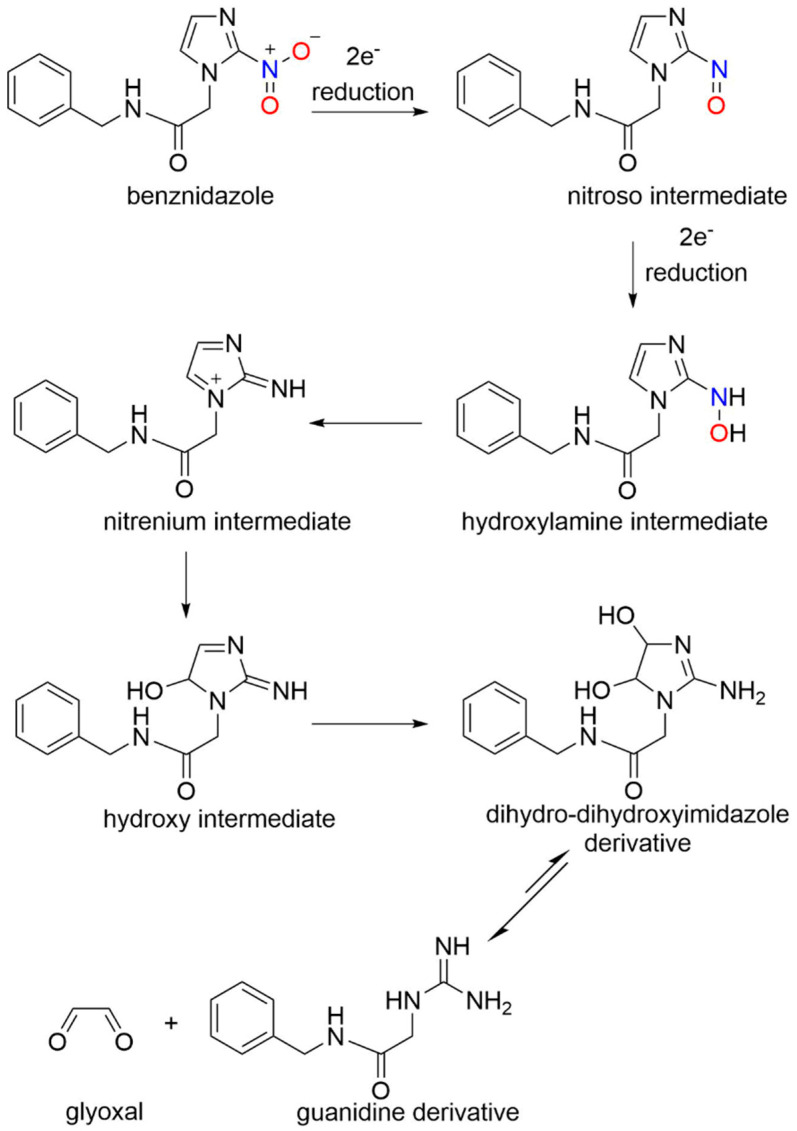
Bioreduction and activation of benznidazole [77].

Benznidazole (*N*-benzyl-2-(2-nitro-1H-imidazol-1-yl)acetamide), a 2-nitroimidazole prodrug, made its debut in the medicinal world in the 1970s [76,77] and is now considered as the current drug of choice to treat Chagas disease (Figure 5). Despite its history and use, the mechanism of benznidazole has remained widely elusive. An NADH-dependent trypanosomal type I nitroreductase plays a crucial step in benznidazole activation [77]. The addition of benznidazole to *T. cruzi* extracts does not increase the production of reactive oxygen species, as previously shown [78], which led to the investigation of an alternative NTR1 mechanism as the cause of trypanocidal activity. After the reduction of benznidazole through NTR1 (Scheme 6), several potentially cytotoxic metabolites were formed that were characterized through liquid chromatography-mass spectrometry (LC-MS) [77]. 

A 4,5-dihydro-4,5-dihydroxyimidazole was identified as the major product of the reductive pathway that passed through nitroso, hydroxylamine and hydroxy intermediates [77]. Ultimate breakdown of the dihydroxy intermediate gives glyoxal, a reactive dialdehyde, which can form adducts with other biological molecules (e.g., guanosine–glyoxal adducts) and a guanidine derivative (Scheme 6) [77]. This process follows the reduction of other 2-nitroimidazoles (Scheme 5) and does not generate nitrogen oxide but could be crucial to the trypanocidal effects of benznidazole [77]. Overall, 2-nitroimidazoles do not generate nitrogen oxides upon reduction but instead fragment to glyoxal and guanidine derivatives that can mediate their biological activity.

### 2.3. 4-Nitroimidazoles

**Figure 6:**
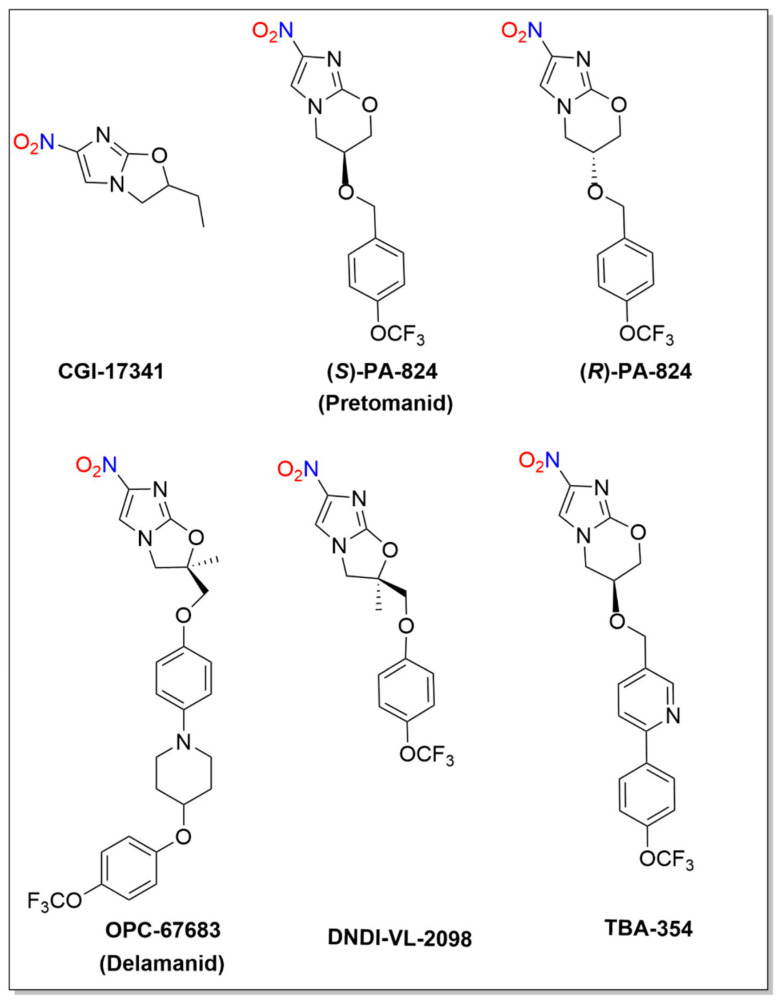
Structures of CG-17341, PA-824, OPC-67683, DNDI-VL-2098 and TBA-354.

Bicyclic 4-nitroimidazoles including CG-17341, PA-824 (Pretomanid), OPC-67683 (Delamanid), DNDI-VL-2098 and TBA-354 demonstrate potent anaerobic activity against non-replicating *Mycobacterium tuberculosis* (MTB) and a variety of anaerobic parasites (Figure 6). Reductive metabolic activation of these compounds generates NO_2_^−^ and/or NO that participate in their biological activity. The Food and Drug Administration (FDA) recently approved Pretomanid tablets, in combination with bedaquiline and linezolid, for treatment of highly treatment-resistant TB [79].

#### 2.3.1. CGI-17341

CGI-17341 served as the lead compound for a 2000 study of a series of 4-substituted bicyclic nitroimidazopyrans (NAPs) in a search for anti-tuberculosis activity [80]. This work identified over 100 compounds possessing either the same or better anti-tubercular activity than CGI-17341 [80]. Structurally, the stereochemistry at the C3 position appears crucial for activity, with the S enantiomer being more active than the R enantiomer by 10-fold [80]. PA-824 emerged from this study showing potent bactericidal activity against MTB with a sub-micromolar MIC, as well as promising oral activity in murine and guinea pig models of infection [80]. The bactericidal activity of PA-824 compares well to metronidazole, while CGI-17341 and the common TB drug isoniazid (INH) were found to be less active against non-replicating MTB [80]. Since metronidazole undergoes nitroreduction for activation against anaerobic bacteria [35], similar metabolic activation pathways for PA-824 were investigated [80]. Incubation of radiolabeled PA-824 with MTB variants revealed metabolic nitroreduction of PA-824 [80]. Mutagenicity issues ultimately stopped the development of CGI-17341 and led to the optimization of other bicyclic nitroimidazoles such as PA-824 (Figure 6) [80].

#### 2.3.2. PA-824 (Pretomanid)

**Scheme 7:**
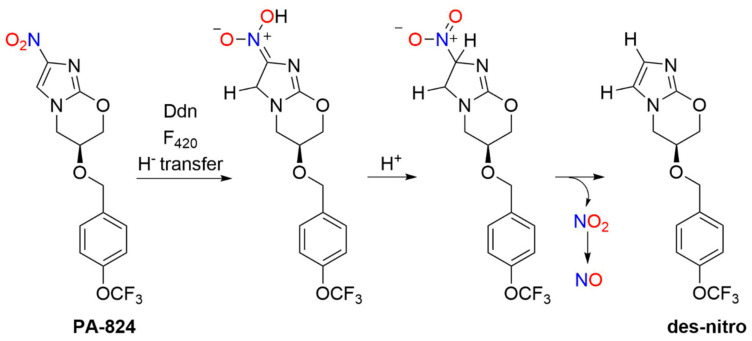
Bioactivation of PA-824 [81].

PA-824, also known by the generic, nonproprietary name Pretomanid, is a novel antibacterial drug in the bicyclic 4-nitroimidazole class of compounds (Figure 6). As mentioned, the FDA approved Pretomanid tablets [79], in combination with bedaquiline and linezolid, for treatment of highly resistant TB in 2019. Like other nitroaromatic antibiotics, PA-824 requires bioactivation and a deazaflavin (F_420_)-dependent nitroreductase (Ddn) catalyzes hydride transfer to PA-824 followed by protonation of the resulting nitronate anion and elimination of nitrous acid to give the des-nitroimidazole (des-nitro) product. Subsequent reduction of NO_2_^−^/nitrous acid provides a pathway (Path A, Scheme 2) to generate reactive nitrogen species, such as NO (Scheme 7) [81].

As a prodrug, PA-824 avoids unwanted drug properties or effects as it only becomes active in bacteria after transformation [82]. A nitro group in the 4-position is crucial for activity as the 5-nitro analog is inactive. The stereochemistry of the side chain appears important for activity, with (*S*)-PA-824 demonstrating superior anti-tubercular activity compared to (*R*)-PA-824 [83]. Replacement of the benzylic oxygen with nitrogen leads to an amine-linked series with favorable in vitro activity and solubility properties [84,85]. In addition, changing the imidazole ring to a pyrazole or triazole maintains activity against MTB, and swapping the oxygen atom in the oxazine ring with sulfoxide, sulfone, amino or methylene groups gives compounds of varying activities [86].

**Scheme 8:**
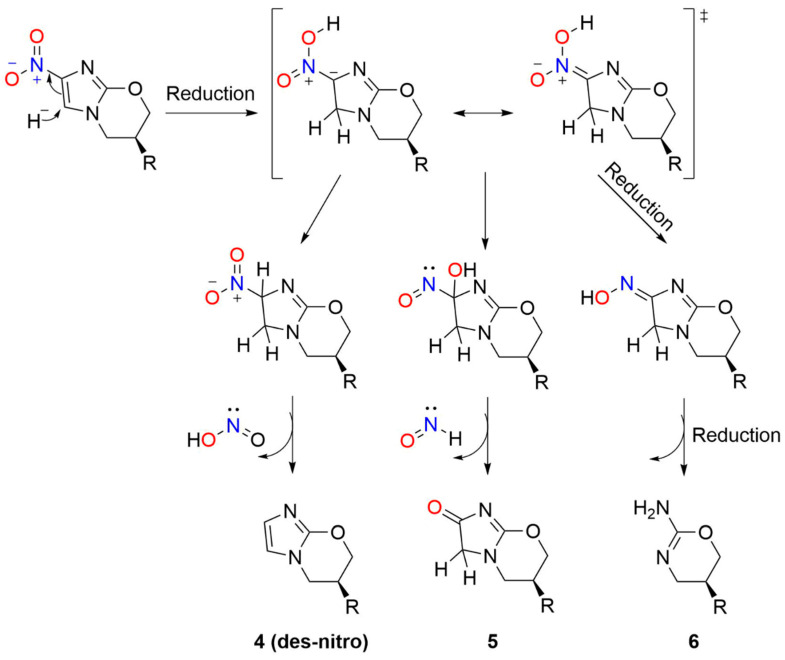
Metabolites of PA-824 [81].

In 2008, Barry and co-workers focused mechanistic studies on the bioactivation of PA-824 and other bicyclic nitroimidazoles for tuberculosis treatment [81]. F_420_H_2_-dependent Ddn-catalyzed bioreduction of (*S*)-PA-824 followed by LC-MS revealed the formation of three primary metabolites (Scheme 8) including the des-nitro product (**4**) as the major metabolite, with smaller amounts of **5** and **6** [81]. Neither the des-nitro metabolite (**4**) nor **5** or **6** possessed any detectable activity against MTB, indicating the importance of the nitro group and its reduction for activity [81]. Chemical reduction of PA-824 with sodium borohydride, as a model of the enzymatic reaction, also yields the des-nitro product (**4**) [81]. Deuterium labeling studies show hydride transfer occurs at the 5-position of the imidazole ring with addition of a proton from the solvent to the original position of the nitro group (4-position) [81].

These observations led to a mechanistic proposal for the formation of **4**–**6** during Ddn-catalyzed reduction of PA-824 [81]. Hydride transfer from F_420_H_2_ to C-5 of the imidazole ring generates a nitronate anion and protonation of this anion followed by elimination of nitrous acid/NO_2_^−^ generates the des-nitro compound (**4**, Scheme 8) [81]. Generation of NO_2_^−^ provides the opportunity for NO formation upon further reduction. Hydrolysis of the nitronate anion (Nef reaction) forms the lactam (**5**) with the elimination of HNO (Scheme 8) [81]. Finally, further nitronate reduction yields **6** (Scheme 8) [81]. The spectrophotometric Griess assay provides evidence of NO_2_^-^ formation during the in vivo incubation of PA-824 with Ddn in the presence of F_420_-H_2_, and fluorescent experiments using diaminofluorescein in whole cells show NO generation [81]. Evidence of HNO was not reported, although the identification of the ultimate organic products may provide information as to the identity of the nitrogen oxide product in these conversions [81]. A series of eight derivatives of PA-824 was studied to correlate NO release with the anaerobic killing ability of MTB and a strong correlation exists between the amount of the des-nitro product formed and killing ability [81]. Addition of C-PTIO ([2-(4-carboxyphenyl)-4,4,5,5-tetramethylimidazoline-1-oxyl-3-oxide]), a specific NO scavenger, decreased the amount of NO formed and decreased PA-824 killing of anaerobic MTB [81]. These results suggest a specific role for NO in the mechanism of action of PA-824 and reveal the mechanism of action of a clinically used TB treatment relies on NO release. [81] Subsequent transcriptional profiling experiments provide further insight about the role of NO in mycobacterial killing by PA-824 by suggesting NO inhibition of cytochrome c oxidase leads to respiratory poisoning under anaerobic conditions [87].

The biochemical properties and catalytic parameters of the deazaflavin (F_420_)-dependent nitroreductase (Ddn) reduction of (*S*)-PA-824 have also been investigated [81]. Monitoring F_420_-H_2_ oxidation and NO_2_^−^ production by UV spectroscopy gives kinetic constants of k_cat_/k_m,app_ = 0.016 μM^−1^ · min^−1^ for (*S*)-PA-824 reduction [81]. Monitoring NO_2_^−^ formation by chemiluminescence NO detection, a more sensitive measure than the Griess assay, yields a k_cat_/k_m,app_ = 0.013 μM^−1^ · min^−1^ using recombinant Ddn expressed and purified as a His6-tagged protein in *Escherichia coli* with F_420_ isolated from *Mycobacterium smegmatis* [81]. Production of NO or HNO or reactions with other biomolecules including F_420_ may account for the observed 10-fold difference in F_420_-H_2_ oxidation and NO_2_^−^ formation [81]. In this system, neither des-nitro PA-824 nor (*R*)-PA-824 supported Ddn-catalyzed oxidation of F_420_-H_2_, indicating these compounds do not act as substrates, supporting previous work that indicates the importance of the nitro group and stereochemistry for activity [81]. Although Ddn does not catalyze (*R*)-PA-824 reduction, fluorescence quenching experiments show (*R*)-PA-824 binds to the enzyme with the same affinity as (*S*)-PA-824, leading to the idea of different binding modes of the enantiomers [81]. Both *R* and *S* enantiomers of CGI-17341 act as Ddn substrates and gave k_cat_/k_m,app_ = 0.011 and 0.020 μM^−1^ · min^−1^, respectively, for F_420_-H_2_ oxidation in this system [88].

The metabolism of PA-824 by mammalian enzymes found in the subcellular fraction of the human liver has also been compared to purified Ddn, and MTB and *M. smegmatis* cellular extracts [89]. Electrospray ionization (ESI) LC-MS analysis confirms that both Ddn and MTB catalyze conversion of PA-824 to the des-nitro compound (**4**), but neither the human liver nor *M. smegmatis* produce **4-6** from PA-824 [89]. The subcellular fraction of the human liver did metabolize PA-824 to four distinct products [89]. Examination through LC-MS indicates the formation of the two-electron imidazole reduction product, also seen in the radiolytic reduction of PA-824 [90], and a hydroxylated imidazole ring product that forms nonenzymatically in the presence of ferrous iron and thiols [89]. The identity of the other two products could not be determined [89]. This study highlights the unlikelihood of metabolic cross-activation of PA-824 and related compounds by mammalian enzymes and their selectivity for organisms capable of hydride transfer (i.e., having a Ddn-type nitroreductase) [89].

Since PA-824 requires reductive bioactivation, electrochemical studies were deployed to better understand the redox properties of PA-824. Even before the mechanism of action for PA-824 was fully determined, the cyclic voltammetric behavior of PA-824 reduction of the nitro group to the corresponding nitro radical anion was examined in comparison to metronidazole [91]. Similar to metronidazole, electrochemical reduction of PA-824 in an aprotic solvent (DMSO) formed a relatively stable nitro radical anion [91]. Formation of the PA-824 radical anion requires more energy for formation (~200 mV), making it less stable (~100x) than the metronidazole radical anion [91]. 

Further work examined the effects of substitution of the imidazole ring of PA-824 on the ease of nitro group reduction and impact on anti-tubercular activity [92]. The 5-position of the imidazole ring was decorated with electron-withdrawing (cyano, chloro and bromo) and electron-donating (amino) substituents and both differential pulse voltammetry and cyclic voltammetry showed their effect on nitro group reduction [92]. Electron-withdrawing groups caused a positive shift and the electron-donating group caused a negative shift in the peak potential value (Ep), which illustrates the tendency of the molecule to be reduced [92]. The cyano derivative showed the greatest shift in peak potential, indicating that its corresponding nitro radical anion would be the most thermodynamically favored [92]. All of the electron-withdrawing substituted derivatives showed a positive shift, but only PA-824 and the cyano derivative exhibited a well-resolved, reversible CV with the cyano derivative nitro radical anion being ~300 times more stable than the PA-824 radical anion [92]. In terms of biological activity, only the bromo and chloro derivatives were active against WT and mutant MTB, suggesting these compounds do not depend on F_420_ Ddn as PA-824, but that reductive bioactivation proceeds through a different pathway, possibly direct reduction of the nitro group [92]. These studies could portend next-generation nitroimidazole derivatives with the alternate nitroreductase pathways. 

The sequential single-electron reduction of PA-824 was investigated through a pulse and steady-state radiolysis study in aqueous solution, which supports a radical mechanism through the novel reduction of the imidazole ring preferentially to the nitro group, unlike other related nitroimidazoles [90]. Stepwise reduction of the compounds was facilitated through the electron transfer from the radical anion of carbon dioxide (CO_2_^•−^). In addition to PA-824, the more soluble compound without the lipophilic sidechain and 2-methoxy-1-methyl-4-nitro-1-*H*-imidazole were studied through UV–VIS spectroscopy, mass spectrometry and DFT calculations, in order to follow the reduction and intermediates [90]. The reduction preference of the nitro group may be due to the 2-alkoxy substitution of the 4-nitroimidazole ring, and the lipophilic side chain of PA-824 could facilitate its ability to bind to a specific protein with the following activation of the 4-nitroimidazole core contributing to the bactericidal ability against MTB [90]. More information is needed to determine if the reduction intermediates play a role in the bactericidal action of PA-824 [90].

More recently, PA-824 was evaluated against a wide range of organisms (e.g., Giardia lamblia, E. histolytica, T. b. brucei, L. donovani, M. tuberculosis, Clostridioides difficile, Cryptococcus neoformans, Candida albicans and representative ESKAPE bacteria: *E. coli*, *Staphylococcus aureus*, *Klebsiella pneumoniae*, *Pseudomonas aeruginosa* and *Acinetobacter baumannii*) [93]. PA-824 showed potent activity against G. lamblia, E. histolytica and C. difficile, which could lead to the expansion of its use as therapy, from Mycobacterium and Leishmania to anaerobic protozoan parasites and anaerobic Gram-positive bacteria [93]. In addition, two novel bicyclic base scaffolds were prepared (e.g., nitroimidazopyrazinones and nitroimidazopyrazines), which were derived from the 4(5)-nitroimidazole scaffold that showed potent activity against M. tuberculosis, G. lamblia and T. b. brucei [93]. Since these organisms do not necessarily possess a deazaflavin-dependent nitroreductase, the identification of the enzymes and mechanisms for the requisite reductive bioactivation may provide leads to the development of new therapies that may include nitrogen oxide release.

#### 2.3.3. (*R*)-PA-824

While (*S*)-PA-824 demonstrates potent activity against MTB, the *R* enantiomer was found to be relatively inactive [81]. Both enantiomers have potent antiparasitic activity against *L. donovani*, the causative agent of VL with (*R*)-PA-824 (Figure 6) being 5-fold more active than (*S*)-PA-824 in Leishmania-infected macrophages [94]. Using a murine model of infection, (*R*)-PA-824 (100 mg/kg) presented an effective cure with 99.9% suppression of infection, while other front-line drugs, sodium stibogluconate and miltefosine, only provided 41.9% and 68.7% suppression of infection, respectively [94]. The nitro group plays a crucial role in the anti-leishmanial activity of PA-824, as both enantiomers of des-nitro PA-824 were inactive against *L. donovani* promastigotes and intracellular amastigotes [94]. Both enantiomers of PA-824 were leishmanicidal in vitro with good selectivity, microsomal stability, aqueous solubility and low plasma protein binding, as well as being orally bioavailable and well tolerated in mice [94]. 

Investigation of the activation of bicyclic nitroaromatic antibiotics (e.g., (*R*)-PA-824, OPC-67683 (vide infra), DNDI-VL-2098 (vide infra) and other related experimental drugs) revealed reduction by a novel NTR2 in Leishmania, which do not contain a F_420_-deazaflavin-dependent nitroreductase [95]. Although monocyclic nitroaromatic antibiotics, such as nifurtimox and benznidazole, are activated by NTR1, quantitative proteomic studies and whole-genome sequencing of susceptible and drug-resistant parasites indicate that NTR2 is necessary and sufficient for the activation of these bicyclic nitroimidazoles through the formation of toxic products, although the direct production of NO from the incubation of either enantiomer of PA-824 to the *Leishmania* NTR2 has not been determined [95]. These studies portend future directions for VL treatment through the intentional repurposing of PA-824, especially in combination with fexinidazole, in antiparasitic therapy.

#### 2.3.4. OPC-67683 (Delamanid) 

OPC-67683 (Delamanid or Deltyba), a bicyclic nitroimidazole, was developed by the Otsuka Pharmaceutical Company, Ltd. and approved by the European Medicines Agency (EMA) in 2014 for the treatment of adult MDR-TB (Figure 6) [96]. OPC-67683 shows potent activity against MTB both in vitro and in vivo in an experimental chronic TB mouse model and MDR-TB with a low MIC [97]. Importantly, OPC-67683 shows a good safety profile in terms of mutagenicity [97]. The good efficacy, lower toxicity and absence of interaction with anti-retroviral drugs suggests use of OPC-67683 for MDR-TB, XDR-TB and TB in HIV-positive patients [98]. In addition, OPC-67683 inhibits VL, both in vitro and in vivo [99].

Similar to PA-824, the mechanism of action of OPC-67683 depends upon reductive activation by the deazaflavin (F_420_)-dependent nitroreductase (Ddn) [88]. OPC-67683 shows substrate-dependent F_420_H_2_ oxidation with purified Ddn, but solubility limitations prevent determination of the kinetic constants for OPC-67683 [88] Ddn-mediated reduction of OPC-67683 generates the corresponding des-nitro compound, which shows no activity against MTB [88]. Intermediates, including NO, formed during this conversion likely mediate the drug’s action by blocking mycolic acid synthesis that disrupts cell wall synthesis, allowing the drug to enter the mycobacteria [88,96].

#### 2.3.5. DNDI Series 

In 2014, DNDI-VL-2098, an OPC-67683 analog, was identified as a preclinical candidate for VL treatment (Figure 6) [100]. A library of 72 compounds was screened for in vitro anti-leishmanial activity against luciferase-transfected DD8 amastigotes of *L. donovani* and DNDI-VL-2098 and its enantiomer, DNDI-VL-2001, were identified as the best candidates [100]. Griess assay analysis verifies NO_2_^−^ and presumably NO generation in hamster splenocytes and extracellular NO generation was significantly higher (>5-fold) when the hamsters were treated with DNDI-VL-2098 in comparison to the infected/untreated group on various days post-treatment [100]. The mechanism of NO release from DNDI-VL-2098 in *L. donovani* has not been elucidated, but the absence of a leishmanial F_420_-deazaflavin-dependent nitroreductase suggests the presence of other bioreductive mechanisms [100]. The idea of NO-mediated suppression of parasites in DNDI-VL-2098-treated hamsters is supported by these studies and provides the basis for NO-based antiparasitic therapies [100]. Additional parallel studies define the essential structural features of DNDI-VL-2098 in terms of activity for VL, solubility and safety [101]. Further studies have led to the development of other potential new leads for VL through the modification of the C-6 position [102].

#### 2.3.6. TBA-354 

TBA-354 is bicyclic 4-nitroimidazole (Figure 6) that initially had great therapeutic promise against TB [103], but the TB Alliance placed a hold on further development in 2016 after clinical trials showed mild signs of neurotoxicity [104]. The mechanism of action of TBA-354 appears similar to PA-824 (vide supra) [105].

## 3. Nitrofurans/Nitrothiophenes 

**Figure 7:**
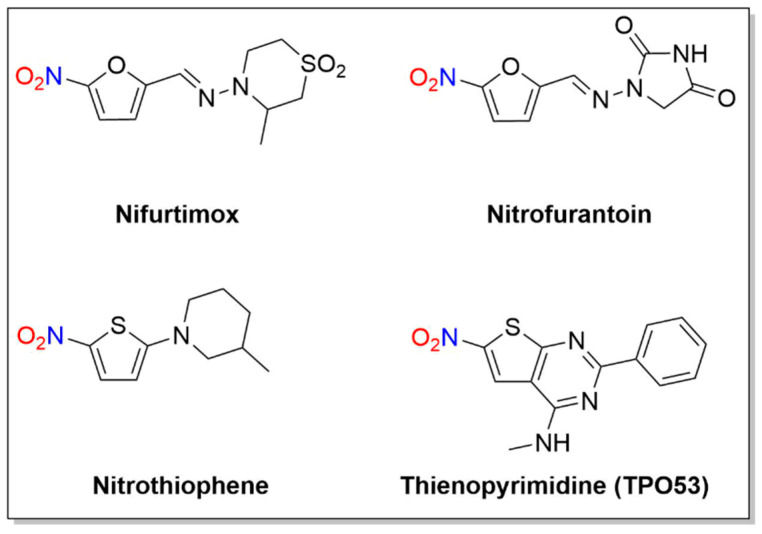
Structures of nifurtimox, nitrofurantoin and thienopyrimidine.

A number of nitro-substituted furans and thiophenes have a long history of use as treatments of anaerobic bacterial infections and parasitic diseases (Figure 7). As with other nitro-substituted aromatic therapeutics, biological activity requires metabolic bioreduction. Early work shows NO_2_^−^ release upon exposure to GSH and liver extracts from some 2-substituted 5-nitrofurans, and NO generation upon chemical reduction of nitro furans has also been reported [44,106]. Representative examples discussed below include nifurtimox, nitrofurantoin and molecules of the nitrothiophene and nitrothienopyrimidine classes (Figure 7). 

### 3.1. Nifurtimox 

Nifurtimox, first introduced in 1965 [107], is a nitrofuran with activity against trypomastigotes and amastigotes and is used to treat Chagas disease, as well as HAT (Figure 7) [107]. 

**Scheme 9:**
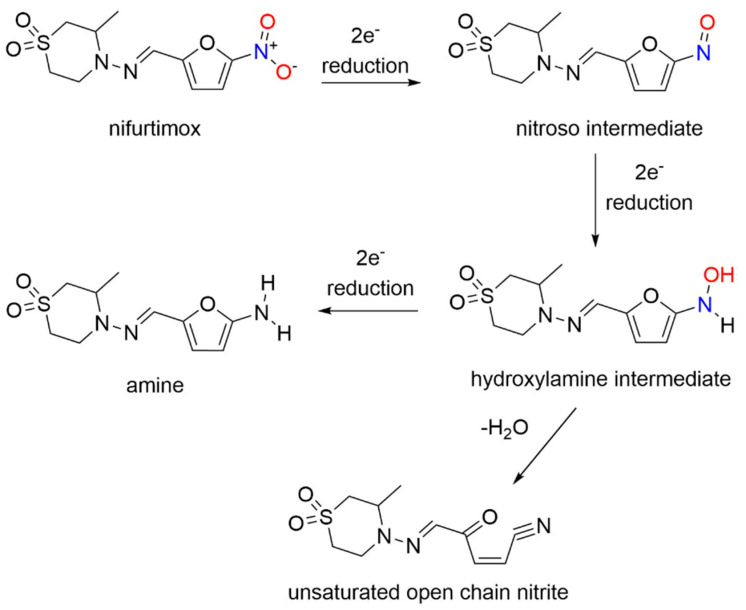
Reduction of nifurtimox [108].

Nifurtimox acts as a prodrug after absorption from the gastrointestinal tract and liver metabolism, where nitroreduction is catalyzed by cytochrome P450 reductase and NTR2, leading to oxidative stress, the generally accepted trypanocidal mechanism of action for nifurtimox [108]. More specifically, nifurtimox metabolism by a trypanosomal NTR1 proceeds through the nitroso and hydroxylamine intermediates, resulting in generation of a cytotoxic ring-opened unsaturated nitrile metabolite as determined by LC-MS (Scheme 9) [108]. 

Unlike nifurtimox, this metabolite inhibits both parasite and mammalian cell growth. This study sheds light on the selective toxicity of nifurtimox as a result of NTR1 activation, which generates secondary toxic organic products rather than nitrogen oxides. 

### 3.2. Nitrofurantoin

Nitrofurantoin, first patented in 1952 [109], is an antibiotic used to prevent and treat bacterial urinary tract infections (e.g., *E. Coli*, *Enterobacter cystitis*, *Enterococcus*, *Klebsiella* and *Staphylococcus aureus*) (Figure 7). The mechanism of action of nitrofurantoin is complex and includes bacterial nitroreductase-catalyzed formation of various reactive intermediates [110]. While reduction of nitrofurantoin generates nitro radical anions in mammalian cells [111], speculation exists whether NO is produced as other early studies found that enzymatic reduction of nitrofurantoin generates ROS [112].

The relationship of NO release and the biological activity of nitrofurantoin was examined on intracellular uropathogenic *E. coli* (UPEC), which causes urinary tract infections (UTI) [113]. Both extracellular and intracellular NO production of infected 5637 bladder cells treated with either the NO donor (2,2′-(hydroxynitrosohydrazono)bis-ethanimine (DETA)/NO)) or nitrofurantoin were measured through either the Griess assay in combination with UV–VIS spectrometry, or DAF-FM-DA and confocal microscopy [113]. While nitrofurantoin was effective against colonized UPEC, neither NO_2_^−^ nor NO was detected in the infected 5637 bladder cells treated with nitrofurantoin through either measurement, indicating that NO is not a key byproduct in the mammalian metabolism of nitrofurantoin [113].

The cytotoxicity of nitrofurantoin in the dark and after light exposure (UVA irradiation, wavelength = 385 nm) was studied in murine melanoma B16F10 cells for a role for NO [114]. Optical absorption and fluorescence spectroscopies, chemiluminescence, fluorescence image microscopy and standard cell viability tests detected NO and its effect on the cells during nitrofurantoin irradiation in both buffered solution and cells [114]. The mechanism of cell death in the dark results from enzymatic NO release, while light exposure increases nitrofurantoin cytotoxicity through increased NO photorelease [114]. The nitrofurantoin cytotoxicity was several times greater upon light exposure [114], which could render this drug a promising photochemotherapy option. 

### 3.3. Nitrothiophenes/Thienopyrimidine (TP053)

Given the similarity of thiophene to furan and imidazole ring systems, a series of 5-nitrothiophenes was evaluated against MTB, revealing a highly active yet non-toxic or mutagenic lead compound (2-(3-methylpiperidin-1-yl)-5-nitrothiophene) from these assays (Figure 7) [115]. Mechanistic studies indicate this 5-nitrothiophene requires the F420-dependent nitroreductase (Ddn) for activity and these compounds generate NO_2_^−^ during incubation with recombinant M. smegmatis containing Ddn [115]. The presence of a nitro group at the C-5 position of the thiophene ring is critical for activity as exchange of the nitro group to an acetyl group or movement of the nitro group from the C-5 to the C-3 position abolishes activity [115]. These compounds appear to act in a nearly identical fashion as the bicyclic 4-nitroimidazoles (e.g., PA-824 and OPC-67683) and their ability to kill MTB and release NO_2_^−^ indicates these thiophene heterocycles can release nitrogen oxides in a similar fashion as the more complicated bicyclic nitroimidazoles, identifying new lead therapeutic compounds [115].

The mechanism of action was studied for the anti-tubercular thienonpyrimidine prodrug (TP053), a fused thiophene–pyrimidine, active against both replicating and non-replicating MTB [116]. Activation of TP053 by a mycothiol-dependent nitroreductase (Mrx2) releases NO and/or NO_2_^−^ as judged by the Griess analysis, similar to PA-824 activation by Ddn [116]. Based on previous studies of 2-nitrofuran reduction [106], a suggested mechanism for NO release involves the conversion of the nitro group (-NO_2_) to a nitrite ester (-ONO) that apparently homolyzes to NO [116]. Further reduction and dearomatization of the thiophene ring gives a thiophenol derivative, as confirmed by LC-MS [116]. This thiophenol (e.g., 2-(4-mercapto-6-(methylamino)-2-phenylpyrimidin-5-yl)ethan-1-ol) contributes to the antimycobacterial effects on both replicating cells and non-replicating cells [116]. These results identify yet another nitroaromatic substrate with a different reducing system capable of producing nitrogen oxides.

### 3.4. 1,3-Benzothiazin-4-ones (BTZs)

#### BTZ043

**Figure 8:**
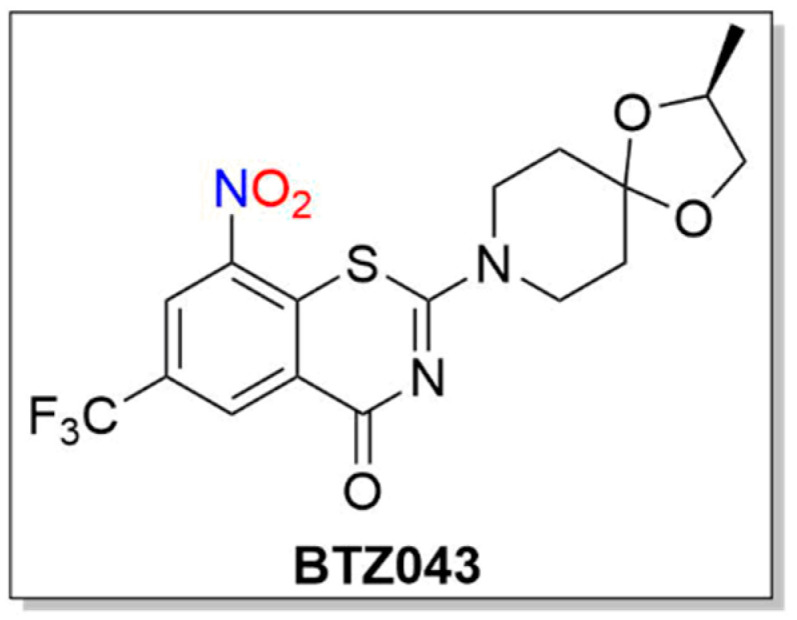
Structure of BTZ043.

Similar to PA-824 and its use as an anti-TB agent, 1,3-benzothiazin-4-ones (BTZs, especially BTZ043) and related nitroaromatic compounds in the nitrobenzothiazione class have emerged as effective candidates for the same purpose (Figure 8) [117].

**Scheme 10:**
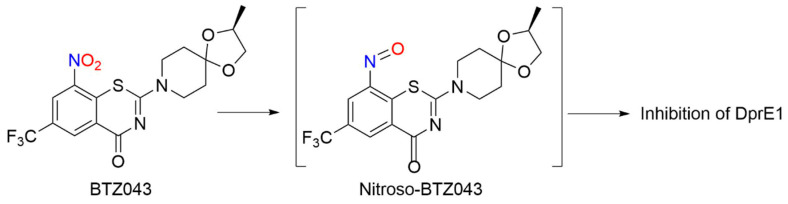
Reduction of BTZ043 [117]^.^

BTZs kill MTB in vitro, ex vivo and in mouse models of TB [118]. Based on potency and specificity for mycobacteria, BTZ0838 (2-[2-methyl1,4-dioxa-8-azaspiro[4.5]dec-8-yl]-8-nitro-6-(trifluoromethyl)-4H-1,3-benzothiazin-4-one)) was synthesized in seven steps, and its enantiomers BTZ043 (*S*) and BTZ044 (*R*) were evaluated in a structure function study [118]. Both the sulfur atom and the nitro group are necessary for activity as chemical reduction of the nitro group to the hydroxylamine/amine diminishes activity. Nitroreductase-producing mycobacteria were resistant to BTZ043 [118]. BTZ043 acts as a prodrug and functions through nitro group reduction to the nitroso, hydroxylamine and ultimately the amine [119]. Both genetic and biochemical studies identify decaprenylphosphoryl-beta-D-ribose 2′-epimerase (DprE) as the target of BTZ [119]. Suicide inhibition of DprE by BTZ043 prevents the formation of decaprenylphosphoryl arabinose, a crucial precursor needed for cell wall arabinan synthesis, leading to death of the bacteria [119]. BTZ043 and nitrobenzamide hybrids react with cysteine thiolates or hydrides at the active site of DprE, inducing the nonenzymatic reduction of the nitro group of BTZ043 to the nitroso intermediate through nucleophilic addition at the unsubstituted electron-deficient aromatic carbon present in these compounds, which leads to inhibition of DprE1 (Scheme 10) [117]. 

This reactivity mirrors the classical von Richter reaction and provides an alternative hypothesis for the mechanism of action for nitroaromatic anti-TB agents, such as BTZ043 [117]. While evidence of NO formation from BTZ043 does not exist or is suggested, these results reveal the rich bio-organic chemistry of these compounds specifically and nitroaromatic antibiotics in general [117]. Based on the promise of BTZs as a novel class of TB drug candidates, a ligand-based design model, based on MIC values from BTZ [118] and predictive solubility, was used to yield a variety of compounds of greater activity than BTZ043 against *M. smegmatis* and MDR-TB with increased solubility, as well as microsomal, metabolic and plasma stability, and low toxicity [120].

## 4. Summary/Perspectives/Future Directions

The presence of a nitro group can be considered a key structural component in certain drugs, as presented herein. Nitroaromatic antibiotics, such as metronidazole, have historically found use in the treatment of anaerobic infections and continue to be developed as therapies (Pretomanid). Reductive metabolic activation provides a unifying feature of these compounds, which act as prodrugs. The requisite reduction of the nitro group leads to a series of reduced reactive intermediates that may directly react with biological targets or fragment into smaller reactive species that elicit biological actions. Reductive metabolic activation of these compounds also provides a mechanism for the generation of reactive nitrogen oxide species including, NO_2_^−^, NO and HNO, which show biological activity. This review specifically highlights the release of nitrogen oxide species from nitroaromatic compounds, which can potentially propel further studies that illustrate the direction of this field, as well as the development of new treatment options. 

As described, the biological reduction of the nitro group of specific drugs results in distinct products. 5-Nitroimidazoles, such as metronidazole and its relatives, fragment to acetamide and oxamic acid derivatives during metabolism with incomplete nitrogen recovery. Nitro group reduction clearly shows NO formation and the direct reaction of thiols with metronidazole reduces the imidazole ring generating NO_2_^−^ directly. 2-Nitroimidazoles, such as benznidazole, form glyoxal and guanidine derivatives upon reduction with no evidence of NO production. Bicyclic 4-nitroimidazoles, such as PA-824, generate NO_2_^-^/NO through direct imidazole ring reduction by a deazaflavin nitroreductase (Ddn). The biological activity of these compounds directly relates to their ability to release NO. Nitrofurans and nitrothiophenes, depending on the structure and biological reductant, either fragment to reactive intermediates or release nitrogen oxides. While reductive metabolic activation unifies these prodrugs, the structure of the compound, the site of reduction (nitro group or aromatic ring), the subsequent pathway (fragmentation/nitrogen oxide release) and the specific reducing systems appear distinct for each drug.

While complex, these various pathways provide an enormous opportunity for the development of selective therapies for tuberculosis and a variety of NTDs, such as HAT, Chagas disease and leishmaniasis. These conditions, as well as many others, urgently need improved treatment options. Evidence presented in this review illustrates that a number of nitroaromatic compounds show activity against various parasites by utilizing the parasite’s distinct metabolic machinery that differs from the host and many bacteria. While development of nitro group-containing compounds has been largely avoided due to their potential mutagenic effects, the potential for the development of specific antibiotics based on the metabolism of a specific organism represents a valuable opportunity. A combination of genetic profiling of target organisms, identification and characterization of potential enzymatic reductants and the design and preparation of new nitro-containing compounds represents a roadmap for developing a new therapeutic that minimizes toxicity issues.

The biological activity of NO_2_^−^, NO and HNO and the observation of nitrogen oxide release from these nitroaromatic antibiotics adds a powerful design element towards new therapeutics. Identification of the reducing system and understanding the mechanism of nitrogen oxide release would permit the incorporation of structural features to ensure release of a specific nitrogen oxide in a controlled manner. Controlled bioactivation of designed nitroaromatic compounds by organism-specific reducing systems holds untapped potential for the delivery of biologically active nitrogen oxide delivery agents with high activity and minimal toxicity.
